# Leukemia cutis als Primärmanifestation einer akuten myeloischen Leukämie

**DOI:** 10.1111/ddg.70004x

**Published:** 2026-06-04

**Authors:** Florian Winkler, Rosa Brand, Dino Mehic, Felix Tuchmann, Teresa Valero, Johanna Strobl

**Affiliations:** ^1^ Universitätsklinik für Dermatologie Medizinische Universität Wien Wien Österreich; ^2^ Klinisches Institut für Pathologie Medizinische Universität Wien Wien Österreich; ^3^ Universitätsklinik für Innere Medizin I Klinische Abteilung für Hämatologie und Hämostaseologie Medizinische Universität Wien Wien Österreich

**Keywords:** Akute myeloische Leukämie, medizinische Dermatologie, Onkologie, Acute myeloid leukemia, medical dermatology, oncology

Sehr geehrte Herausgeber,

Wir berichten über den Fall eines 60‐jährigen Mannes, der sich in der Ambulanz der Universitätsklinik für Dermatologie der Medizinischen Universität Wien vorstellte. Er präsentierte sich mit einem generalisierten Hautausschlag und starkem Juckreiz, welche seit 2 Monaten bestanden. Zuvor war er mit topischen Kortikosteroiden, systemischen Antihistaminika und systemischer Antibiose behandelt worden, ohne dass eine Besserung eintrat. Im dermatologische Status zeigte sich ein generalisiertes Exanthem mit erythematösen, flachen und polygonalen Papeln, die teils zu Plaques konfluierten, sowie lineare Exkoriationen und Krusten (Abbildung 1a).

**ABBILDUNG 1:**
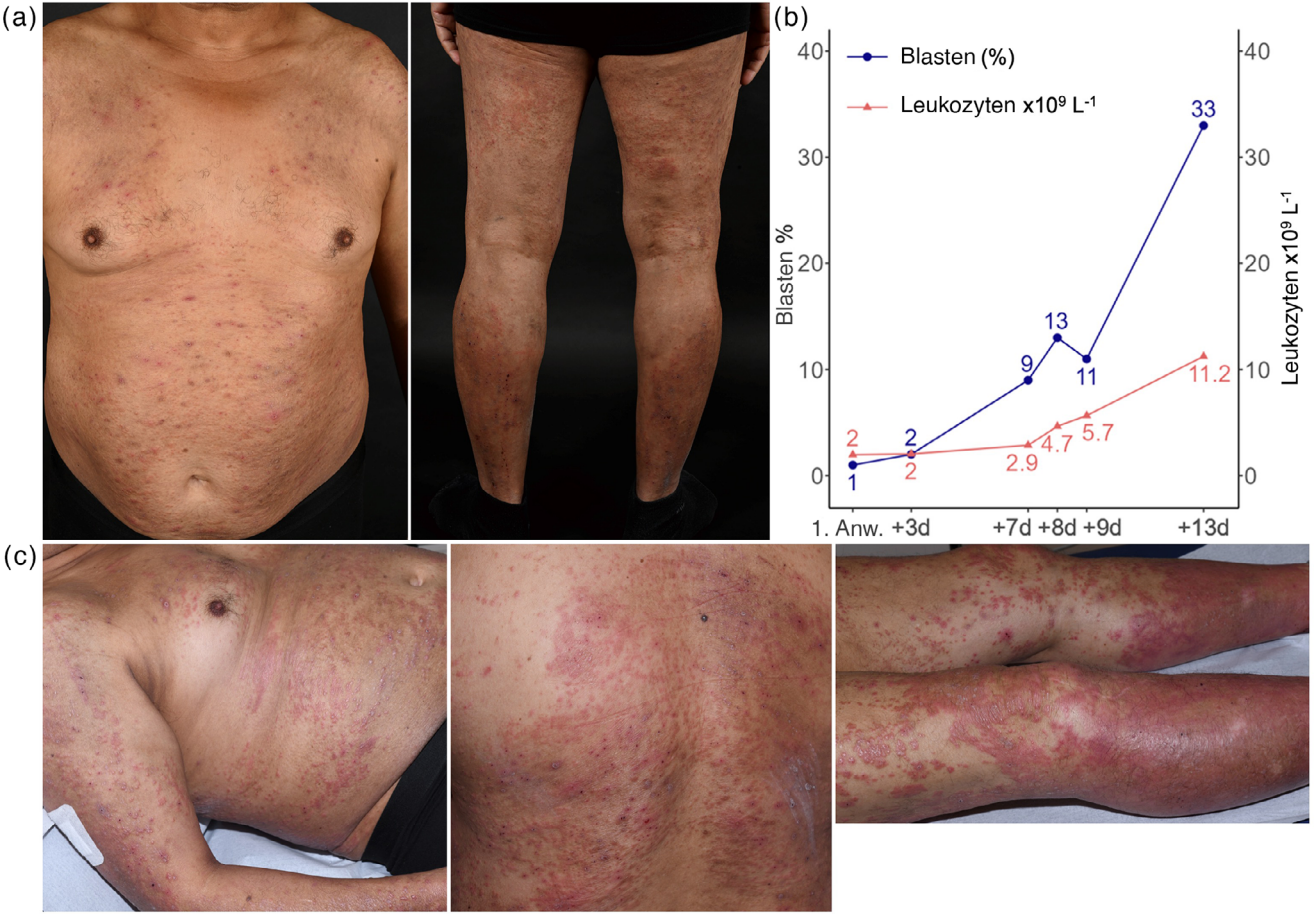
Klinisches Erscheinungsbild. (a) Initiale klinische Präsentation in unserer Ambulanz. (b) Zeitlicher Verlauf des Blastenanteils (Prozent der Leukozyten) sowie der Leukozytenzahl im peripheren Blut. (c) Klinisches Erscheinungsbild 7 Tage nach der initialen Präsentation unter lokaler Therapie mit Clobetasolpropionat 2‐mal täglich.

Die erste Untersuchung des peripheren Blutes zeigte eine Leukopenie von 1,97 G/l (× 10^9^/l, Referenzbereich 4000–10 000 Zellen/µl), einen Hämoglobinspiegel von 12,3 g/dl (13,5–18,0 g/dl) und eine normale Thrombozytenzahl von 194 G/l (150–350 G/l). Das Differenzialblutbild ergab 36 % segmentierte Neutrophile (50–75 %), 4 % stabförmige Neutrophile (3,0–5,0 %), 46 % Lymphozyten (25,0–40,0 %) und 1 % Blasten, ohne Myelozyten oder Metamyelozyten. Nach der Durchführung einer Hautbiopsie wurde der Patient lokal mit Clobetasolpropionat zweimal täglich behandelt. Eine Woche nach der Erstvorstellung schritt das Exanthem trotz lokaler Therapie fort (Abbildung 1c), und der Patient wurde zur Durchführung einer intensivierten Lokaltherapie und weiterer Untersuchungen stationär aufgenommen.

Die nachfolgenden Laboruntersuchungen zeigten einen allmählichen Anstieg der Leukozyten und zirkulierenden Blasten, mit 33 % Blasten im Differenzialblutbild 13 Tage nach der Erstvorstellung (Abbildung 1b). Immunphänotypisch waren die Blasten positiv für CD117, negativ für CD34 und zeigten eine relativ starke Expression von CD123 und CD33. Die Hautbiopsie zeigte eine dichte Infiltration mit CD33‐positiven Zellen und Zellen mit zytoplasmatischem Nucleophosmin (NPMcy^+^), was auf die Diagnose Leukemia cutis hindeutete. Eine weitere immunhistochemische Analyse ergab keine Reaktion mit CD34 und eine partielle Reaktion mit CD117 und Myeloperoxidase (MPO) (Abbildung 2a). Die in Folge durchgeführte Knochenmarksbiopsie bestätigte die Diagnose einer akuten myeloischen Leukämie (AML) und zeigte eine Infiltration mit NPMcy^+^‐Blasten, die in der Chloracetatesterase‐Färbung (CAE) negativ blieben (Abbildung 2b). Die diagnostischen Implikationen der in Abbildung 2 dargestellten immunphänotypischen Marker sind in Tabelle 1 aufgeführt.

**ABBILDUNG 2:**
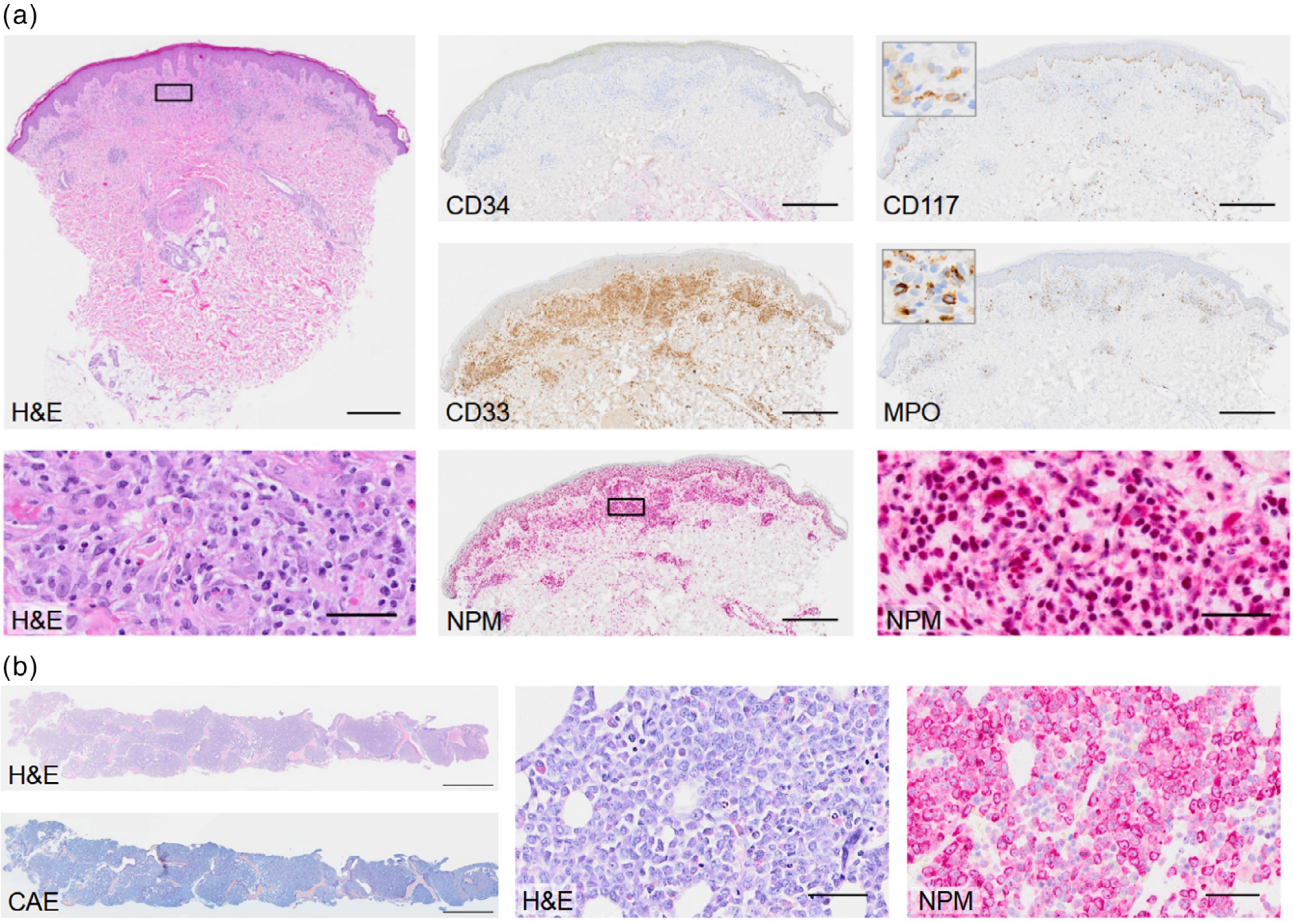
Hautinfiltration mit NPMcy^+^ AML‐Zellen. (a) Perivaskuläre, fleckige gemischte Zellinfiltrate in der oberflächlichen Dermis, bestehend aus Lymphozyten, Histiozyten, eosinophilen Granulozyten und Blasten mit lockerer Chromatinstruktur und feinen Nukleoli. Die Immunhistochemie (IHC) zeigt eine starke Reaktion mit CD33 sowie eine partielle Reaktion mit CD117 und Myeloperoxidase (MPO), während CD34 keine Reaktion zeigt. Zusätzlich zur physiologischen Kernpositivität zeigt Nucleophosmin (NPM) eine zarte zytoplasmatische (cy) Färbung. Maßstäbe: 500 µm (Übersichten) und 50 µm (Vergrößerungen). (b) Ausgeprägte Knochenmarkinfiltration durch NPMcy^+^ AML. Die Blasten bleiben in der Chloracetatesterase (CAE)‐Färbung ausgespart; eine zusätzliche IHC ergab folgendes Expressionsprofil: CD117^+^, MPO^+^, CD33^+^, CD4^+/−^, CD11c^+/−^, CD7^+/−^, CD34^−^ (Daten nicht gezeigt). Maßstäbe: 2 mm (Übersichten) und 50 µm (Vergrößerungen).

**TABELLE 1:** In Abbildung 2 dargestellte immunphänotypische Marker und ihre diagnostische Bedeutung.

Immunphänotypische Marker	Diagnostische Implikationen
NPMcy	Hinweis auf eine *NPM1*‐Mutation, günstige Risikogruppe gemäß Risikostratifizierung des *European LeukemiaNet* (ELN) 2022. Schlechtere Prognose bei gleichzeitiger FLT‐ITD‐Mutation, mittleres Risiko gemäß ELN.^1^
CD34 CD117	Marker, die auf niedrig differenzierte Blasten hinweisen, die aus einem frühen Vorläuferstadium hervorgehen.^1^ CD117 und CD34 wurden in Myeloblasten im Vergleich zu normalen myeloischen Vorläuferzellen in höherem Maße exprimiert und könnten daher als potenzielle diagnostische Marker für den Nachweis minimaler Resterkrankungen bei AML dienen.^2^
MPO	Marker, der bestätigt, dass die Blasten myeloischen Ursprungs sind.^1^
CD33	Myeloischer Marker.^1^ Therapieoption mit dem CD33‐Inhibitor Gemtuzumab Ozogamicin.^3^
CD123	Leukämie‐Stammzellmarker.^1^ Ziel von experimentellen Therapien, wie beispielsweise gegen CD123 gerichtete chimäre Antigenrezeptor (CAR)‐T‐Zell‐Therapien.^4^
CAE	Marker für myeloische und granulozytäre Vorläuferzellen, Unterscheidung zwischen monozytärer und granulozytärer AML.^5^

Die genetische Untersuchung ergab eine *FLT3*‐ITD‐Mutation, ohne dass andere Mutationen oder Chromosomenanomalien nachweisbar waren, insbesondere keine *NPM1*‐Mutation. Während NPMcy als Surrogatmarker für die *NPM1*‐Mutation dient, kann eine NPMcy‐Positivität in seltenen Fällen auch ohne *NPM1*‐Mutation auftreten. Aufgrund der genetischen Analyse wurde der Patient gemäß der Risikostratifizierung des *European LeukemiaNet* (ELN) aus dem Jahr 2022 der Kategorie mit mittlerem Risiko zugeordnet. Anschließend erhielt der Patient eine Induktionschemotherapie mit 7 Tagen Cytarabin und 3 Tagen Daunorubicin. Er erreichte eine komplette hämatologische Remission, die mit einer vollständigen Rückbildung der Hautveränderungen einherging. Der Patient erhielt anschließend vier Zyklen einer Konsolidierungstherapie mit einem Cytarabin‐haltigen Schema und eine Erhaltungstherapie mit Zugabe des FLT3‐Inhibitors Midostaurin, da eine *FLT3*‐ITD‐Mutation nachgewiesen wurde, die zu einer konstitutiven Aktivierung der Rezeptortyrosinkinase FLT3 führt, was eine erhöhte Proliferation von hämatopoetischen und leukämischen Zellen zur Folge hat. Leider kam es nach der Behandlung zu einem Rezidiv der Grunderkrankung, an der der Patient etwa ein Jahr nach der Erstdiagnose verstarb.

Hier berichten wir über einen Patienten, dessen generalisiertes papulöses Exanthem letztendlich zur Diagnose einer AML führte, einer hämatopoetischen Neoplasie, die durch eine unkontrollierte Proliferation unreifer myeloischer Zellen gekennzeichnet ist.^6^, ^7^ Zusätzlich zu den Symptomen, die auf qualitativen und quantitativen Zellabweichungen beruhen, zeigen Leukämien gelegentlich Anzeichen einer extramedullären Infiltration.^8^ Eine solche Manifestation ist die Leukemia cutis, eine Erkrankung, bei der leukämische Zellen in die Haut eindringen und zur Ausbildung von Hauteruptionen führen.^9^, ^10^ Rezente Studien haben gezeigt, dass Leukemia cutis bei AML‐Patienten mit einem ungünstigen Verlauf verbunden ist.^11^, ^12^ Eine Kohortenstudie zeigte eine niedrigere 5‐Jahres‐Überlebensrate von 8,6 % bei AML‐Patienten mit durch Biopsie bestätigter Leukemia cutis, im Vergleich zu 28,3 % bei AML‐Patienten ohne Leukemia cutis, was darauf hindeutet, dass Leukemia cutis Patienten möglicherweise eine aggressivere Therapie benötigen.^11^ Die therapeutischen Möglichkeiten haben sich in den letzten Jahren stetig erweitert. Unser Patient wies eine *FLT3*‐ITD‐Mutation auf, die mit einer verminderten Gesamtüberlebensrate assoziiert und Gegenstand der Entwicklung gezielter Therapien ist.^13^, ^14^ Tatsächlich konnten mehrere Studien zeigen, dass die Zugabe von FLT3‐Inhibitoren (zum Beispiel Midostaurin, Gilteritinib) zu einer Chemotherapie zu einer verbesserten Überlebensrate bei AML‐Patienten mit einer FLT3‐ITD‐Mutation führte.^13^, ^15^, ^16^ Eine retrospektive Studie von Bazinet et al. zeigte, dass AML‐Patienten, die mit einem FLT3‐Inhibitor behandelt wurden, ein signifikant längeres rezidivfreies Überleben von 32,3 Monaten und ein Gesamtüberleben von 35,5 Monaten erreichten, verglichen mit 14,3 beziehungsweise 18,9 Monaten bei Patienten, die keinen FLT3‐Inhibitor erhielten.^13^ Bei Patienten, die für eine intensive Chemotherapie nicht in Frage kommen, verbesserte die Kombination einer niedrigintensiven Therapie mit einem FLT3‐Inhibitor und dem BCL‐2‐Inhibitor Venetoclax das Gesamtüberleben im Vergleich zu anderen Behandlungskombinationen signifikant auf 19,1 Monate, was Dreifach‐Therapien zu einem vielversprechenden Ansatz bei Patienten macht, die für eine intensive Chemotherapie nicht geeignet sind.^13^, ^17^ Leukemia cutis wurde in einer Querschnittstudie bei 3,7 % der Patienten mit AML diagnostiziert, wobei 2,9 % bioptisch bestätigt wurden.^18^ In der Literatur traten die meisten Fälle von Hautläsionen nach oder während der Entwicklung der Bluterkrankung auf. Nur wenige Fälle wurden beschrieben, in denen die Hautveränderungen dem Ausbruch der Bluterkrankung vorausgingen.^19^, ^21^ Aktuelle Literaturfälle von Leukemia cutis sind in Tabelle 2 aufgeführt.

**TABELLE 2:** Aktuelle Literaturfälle von Leukämie cutis aus den Jahren 2023–2025.

Referenz	Alter und Geschlecht	Mutation	Latenz zur Diagnose AML	Prognose
Buzzatti et al.^22^	58 Jahre, weiblich	*NPM1*, Isocitrat‐Dehydrogenase1 (IDH1)	Begleitend zur Diagnose der AML	Rezidivfreies Überleben 11 Monate nach Beginn der Behandlung
Nahm et al.^23^	59 Jahre, weiblich	*FLT3* ITD, *FLT3* D835	Begleitend zum Rediziv der AML, 3 Jahre nach der Erstdiagnose	Patientin 7 Monate nach Rezidiv der Erkrankung verstorben
Gayibov et al.^24^	36 Jahre, weiblich	Nicht angegeben	Leukemia cutis ging einem systemischen Rezidiv der AML voraus	Patientin 3 Monate nach Rezidiv der Erkrankung verstorben
Shen et al.^25^	43 Jahre, weiblich	Nicht angegeben	Keine systemische Erkrankung innerhalb von 8 Monaten klinischer Verlaufskontrollen	Vollständige Rückbildung der Hautläsionen, allogene Stammzelltransplantation zum Zeitpunkt des Berichts geplant
Selan et al.^26^	70 Jahre, männlich	*NPM1*	Begleitend zur Diagnose der AML	Patient 4–5 Monate nach Diagnosestellung verstorben
Mandal et al.^27^	22 Jahre, weiblich	Nicht angegeben	Hautveränderungen bestanden einen Monat vor der Diagnose der AML	Nicht angegeben
Taha et al.^28^	86 Jahre, männlich	Nicht angegeben	Keine Anzeichen einer systemischen Erkrankung zum Zeitpunkt des Berichts	Nicht angegeben
Niscola et al.^29^	81 Jahre, männlich	*NPM1*	Begleitend zur Entwicklung von AML aus einem myelodysplastischen Syndrom	Komplette hämatologische und kutane Remission 18 Monate nach der Diagnose der AML

Gemäß der Eigenanamnese waren die Hautläsionen des präsentierten Patienten bereits zwei Monate vor der Erstvorstellung in unserer Ambulanz vorhanden, und frühere Behandlungsversuche hatten zu keiner Besserung geführt. Aufgrund des geringen Anteils an Blasten zum Zeitpunkt der Erstvorstellung und der Tatsache, dass die Hauterkrankung bereits seit einiger Zeit bestand, kann davon ausgegangen werden, dass die Hautveränderungen der floriden hämatologischen Erkrankung vorausgingen. Die Haut ist selten der primäre Manifestationsort einer AML, was die Frage nach den pathogenetischen Mechanismen aufwirft, durch die Leukämiezellen primär die Haut infiltrieren, und wie sich diese Zellen von Leukämiezellen unterscheiden, die nicht zu einer extramedullären Infiltration führen. Eine mögliche Erklärung bietet die Funktion von Chemokinrezeptoren und Adhäsionsmolekülen.^12^ Die leukämische Hautinfiltration könnte auf eine abnorme Expression von Skin‐Homing‐Chemokinrezeptoren wie CCR4 und CXCR4 und Adhäsionsmolekülen wie CD56 und ICAM‐1 zurückzuführen sein.^30^ Immunhistochemische Studien zeigten eine diskordant höhere Expression von CD56 und eine geringere Expression von CD117 und CD34 bei myeloischer Leukämie der Haut im Vergleich zum Immunphänotyp der Blasten im Knochenmark.^31^ Zusätzlich zu einer möglichen Rolle von Adhäsionsmolekülen und Chemokinrezeptoren sind verschiedene Mutationen in den Blasten mit einer Tendenz zur extramedullären Infiltration der letzteren verbunden. Tatsächlich ist die *FLT3*‐Mutation mit einer extramedullären Infiltration bei AML‐Patienten verbunden^32^ und korreliert nachweislich positiv mit der CXCR4‐Expression.^33^ Weitere Mutationen, die mit extramedullären Manifestationen assoziiert sind, betreffen die Gene *NPM1* und *KMT2A*, die mit einem monozytären Phänotyp der Leukämiezellen in Verbindung stehen.^11^, ^24^ Dies steht im Einklang mit der Erkenntnis, dass insbesondere AML mit monozytärer und myelomonozytärer Differenzierung mit extramedullären Manifestationen assoziiert ist.^18^

Aufgrund des heterogenen klinischen Erscheinungsbildes und der klinischen Ähnlichkeit mit anderen Hauterkrankungen kann die Diagnose einer aleukämischen Leukemia cutis schwierig sein. Im vorliegenden Fall gehörten ein generalisierter Lichen planus, Arzneimittelexanthem, primäres kutanes Lymphom oder eine Pityriasis lichenoides et varioliformis acuta (PLEVA) zu den Differenzialdiagnosen bei der Erstvorstellung. Leukemia cutis ist in der Regel durch einzelne oder multiple, sich schnell entwickelnde Papeln, Knötchen oder Plaques an den Extremitäten, am Rumpf, auf der Kopfhaut oder im Gesicht gekennzeichnet.^34^, ^35^ Die klinische Morphologie ermöglicht die Unterscheidung von Lichen planus (polygonale, flache Papeln mit Wickham‐Streifen und Lokalisation an Extremitätenstreckseiten; Schleimhautbeteiligung)^36^ und PLEVA (Papeln, die sich zu Erosionen, Ulzera und hämorrhagischen Krusten entwickeln).^37^ Die Anamnese einschließlich Medikamentenexposition und Dauer der Läsionen kann Arzneimittelexantheme und kutane T‐Zell‐Lymphome differenzieren.^38^, ^39^ Ein strukturierter Algorithmus für die Diagnose von Leukemia cutis und die Unterscheidung wichtiger Differenzialdiagnosen mit Schwerpunkt auf histologischen und immunhistochemischen Analysen wurde von Jenei et al. vorgeschlagen.^35^ Der vorliegende Fall unterstreicht die Bedeutung der Berücksichtigung einer malignen hämatologischen Erkrankung in der Differenzialdiagnose eines therapieresistenten Exanthems. Angesichts der schlechten Prognose bei Leukemia cutis ist eine frühzeitige Biopsie mit erweiterter Immunphänotypisierung für eine rechtzeitige Diagnose und adäquate Therapie unerlässlich.

## DANKSAGUNG

Open access funding provided by Medizinische Universität Wien/Kooperation E‐Medien Österreich (KEMÖ).

## INTERESSENKONFLIKT

Keiner.
